# An unusual case of infective spondylodiscitis caused by *Campylobacter fetus* subsp. *fetus*: molecular characterization by whole-genome sequencing

**DOI:** 10.1099/acmi.0.000240

**Published:** 2021-07-08

**Authors:** Dhiviya Prabaa MS, Jaya Lakshmi SS, John Antony Jude Prakash, Kenny S. David, Vijay Alagar, Balaji Veeraraghavan

**Affiliations:** ^1^​ Department of Clinical Microbiology, Christian Medical College, Vellore 632004, India; ^2^​ Department of Spine Surgery, Christian Medical College, Vellore 632004, India

**Keywords:** spondylodiscitis, *C. fetus*, MALDI-TOF, MRI, whole-genome sequencing

## Abstract

Spondylodiscitis is an infectious inflammation that affects the intervertebral disc and adjacent structures. Treating infective spondylodiscitis is often challenging due to the lack of specific symptoms. Here we present an unusual case of infective spondylodiscitis caused by *Campylobacter fetus* subsp. *fetus*.

## Introduction

*Campylobacter* is a foodborne zoonotic pathogen and one of the common causes of bacterial diarrhoea in both developed and developing countries [1]. *Campylobacter jejuni* and *Campylobacter coli* are a major cause of gastroenteritis in humans [2]. The species *Campylobacter fetus* can cause disease in both humans and animals. Nearly all *C. fetus* infections in humans are reported to be caused by *C. fetus* subsp. *fetus* [3]. In humans, *C. fetus* infections vary from acute diarrhoea to systemic illness, mainly affecting elderly and immunocompromised individuals [4]. Further, humans show a lack of long-term immunity, as *Campylobacter*-specific antibodies may decrease within a few months after a single enteritis episode [5].

Infective spondylodiscitis is the infection of the vertebral body, its posterior arch and the intervening disc, with hospital mortality rates of 2–17 % [6]. The majority of the cases involve the vertebral body and/or the intervertebral disc, while involvement of the posterior elements of the spine is seen only in 5 % of cases. Spondylodiscitis can be pathologically divided into two broad categories, acute pyogenic and chronic [6]. The most common pathogen reported to cause pyogenic spondylodiscitis is *Staphylococcus aureus*, which accounts for 20–84 % of non-tuberculous cases. It is rarely caused by *Campylobacter* species. Here we report a case of infective spondylodiscitis caused by *C. fetus* subsp. *fetus* for the first time in India, and share our experience of this rare occurrence to improve patient outcomes in the future.

## Case report

**Fig. 1.**
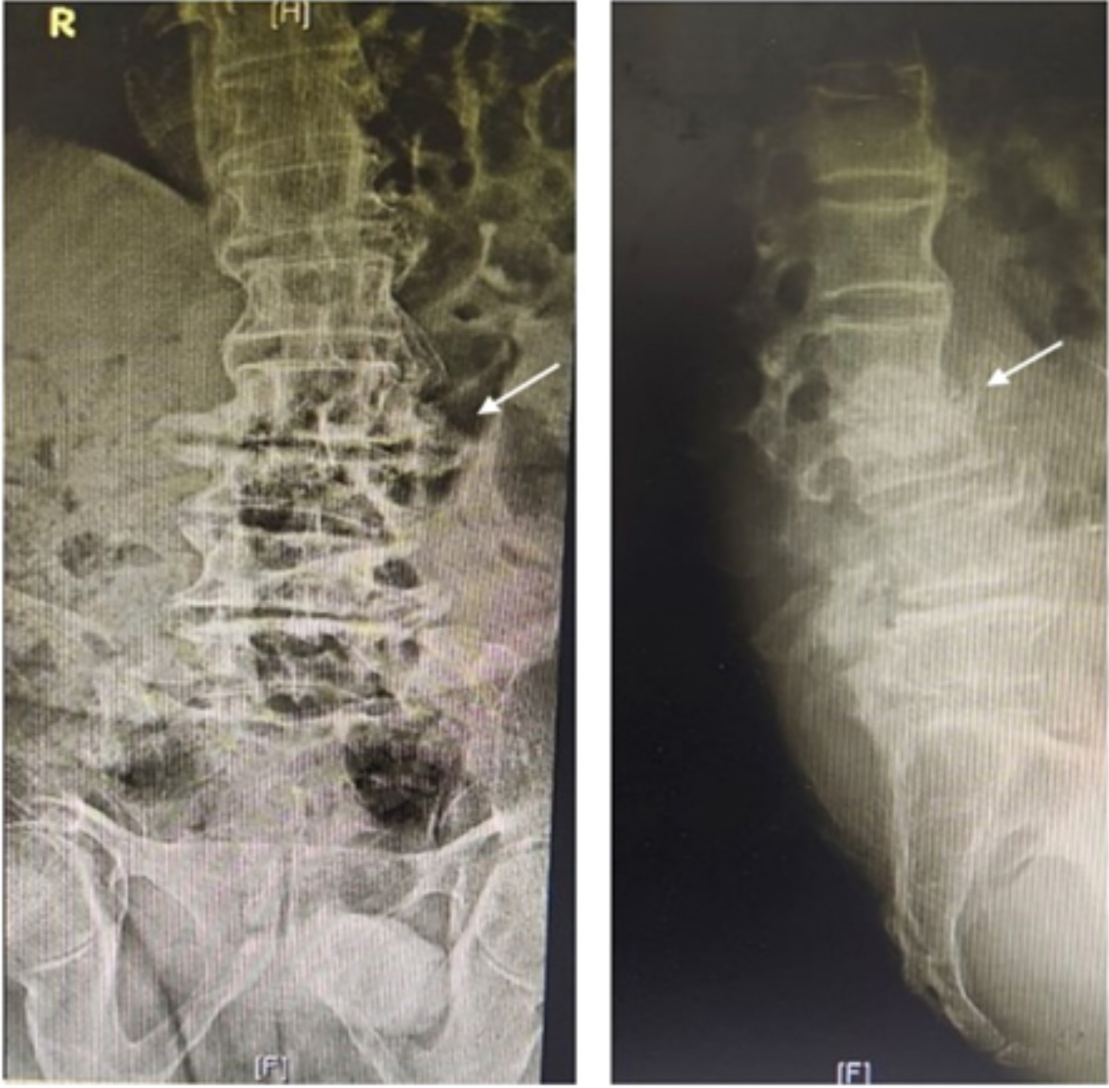
Lumbar spine radiographs (AP and lateral views) showing collapse of the L2–3 disc space with local kyphosis, apart from multilevel degenerative changes.

**Fig. 2.**
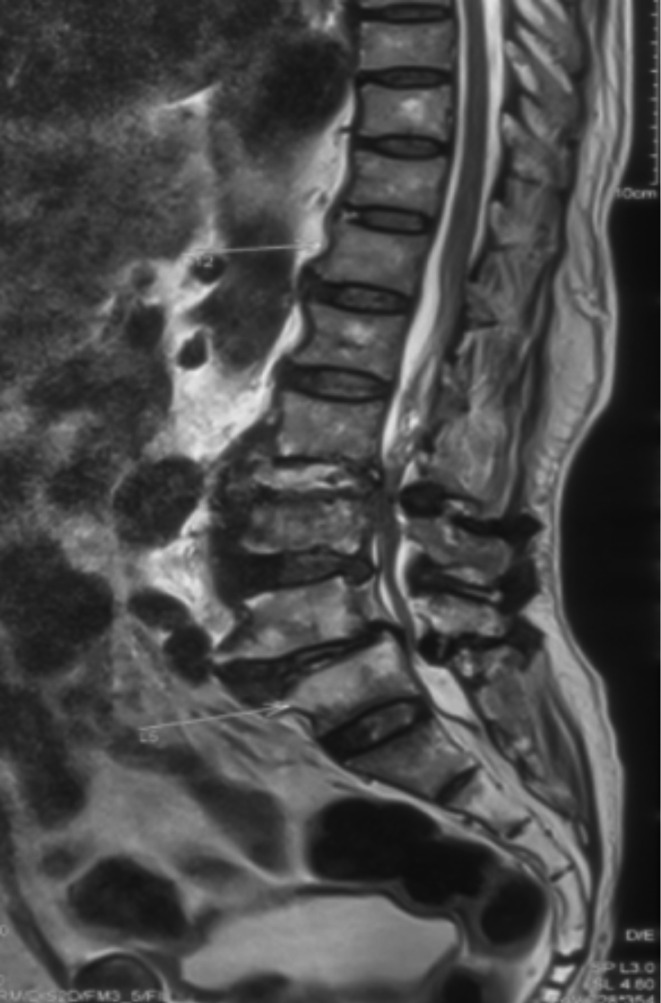
Magnetic resonance imaging T2 sagittal image showing features suggestive of spondylodiscitis at L2–3.

**Fig. 3.**
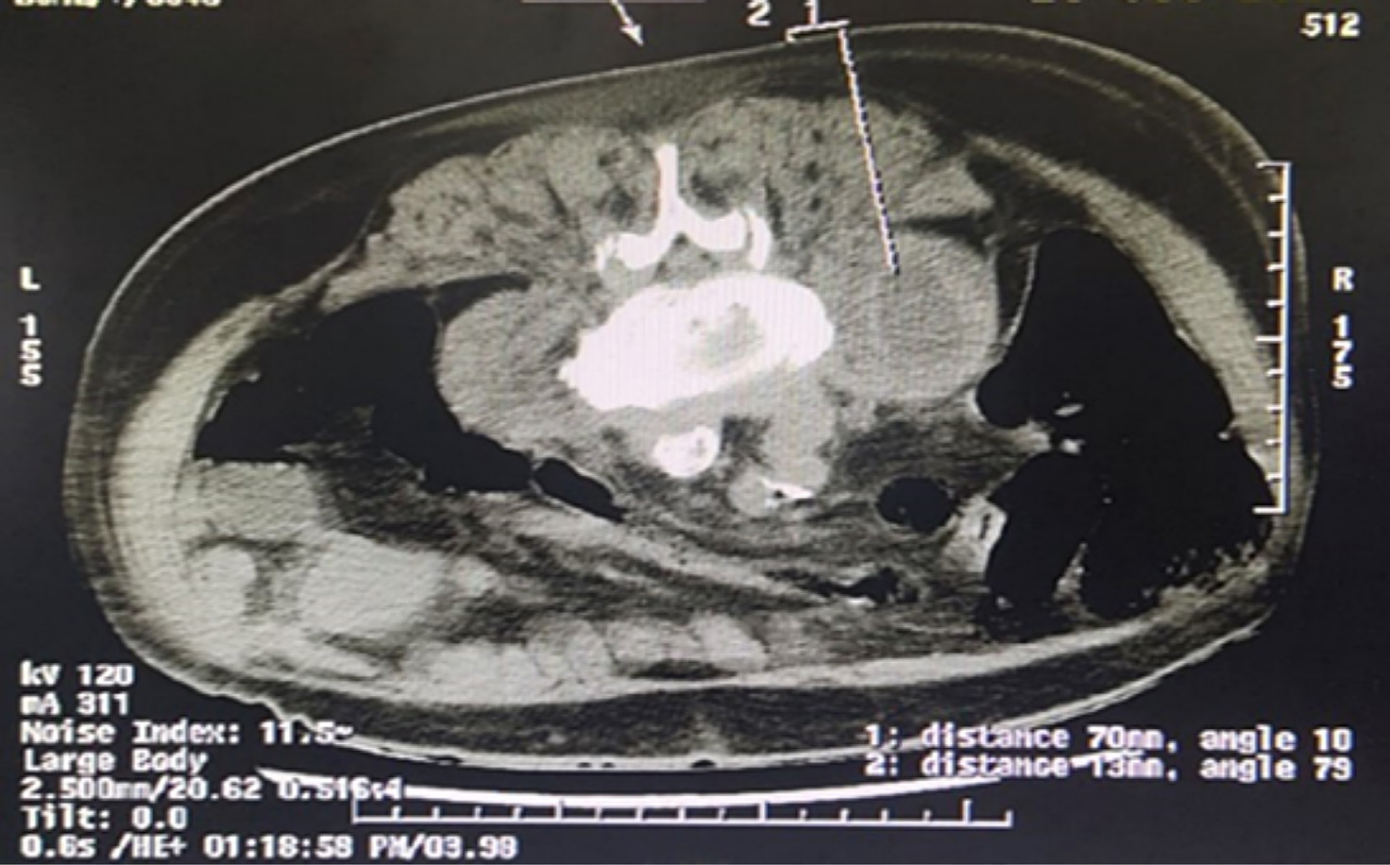
CT-guided aspiration/biopsy of the right-sided psoas abscess.

We describe a 73-year-old patient who presented with progressively increasing lower back pain for the previous 1 month and fever for the last 1 week in October 2019. The medical history included surgery for coronary artery disease 13 years previously and hypertension under treatment. Radiographs showed multilevel degeneration in the lumbar spine and collapse of the L2–3 disc space with some local kyphosis (Fig. 1). Lumbar magnetic resonance imaging (MRI) showed paradiscal signal changes at L2–3 consistent with infection, in addition to a right-sided psoas abscess (Fig. 2). A clinical diagnosis of infective spondylodiscitis was made, and a CT-guided biopsy was performed (Fig. 3). The material was sent for aerobic and anaerobic culture. Further, Gene Xpert TB PCR was negative for *Mycobacterium tuberculosis*, while aspirate smears were also negative for acid-fast bacilli (AFB). Additionally, investigation of inflammatory markers showed C-reactive protein of 126 mg l^−1^, which is significantly higher than the normal level (<6 mg l^−1^).

**Fig. 4.**
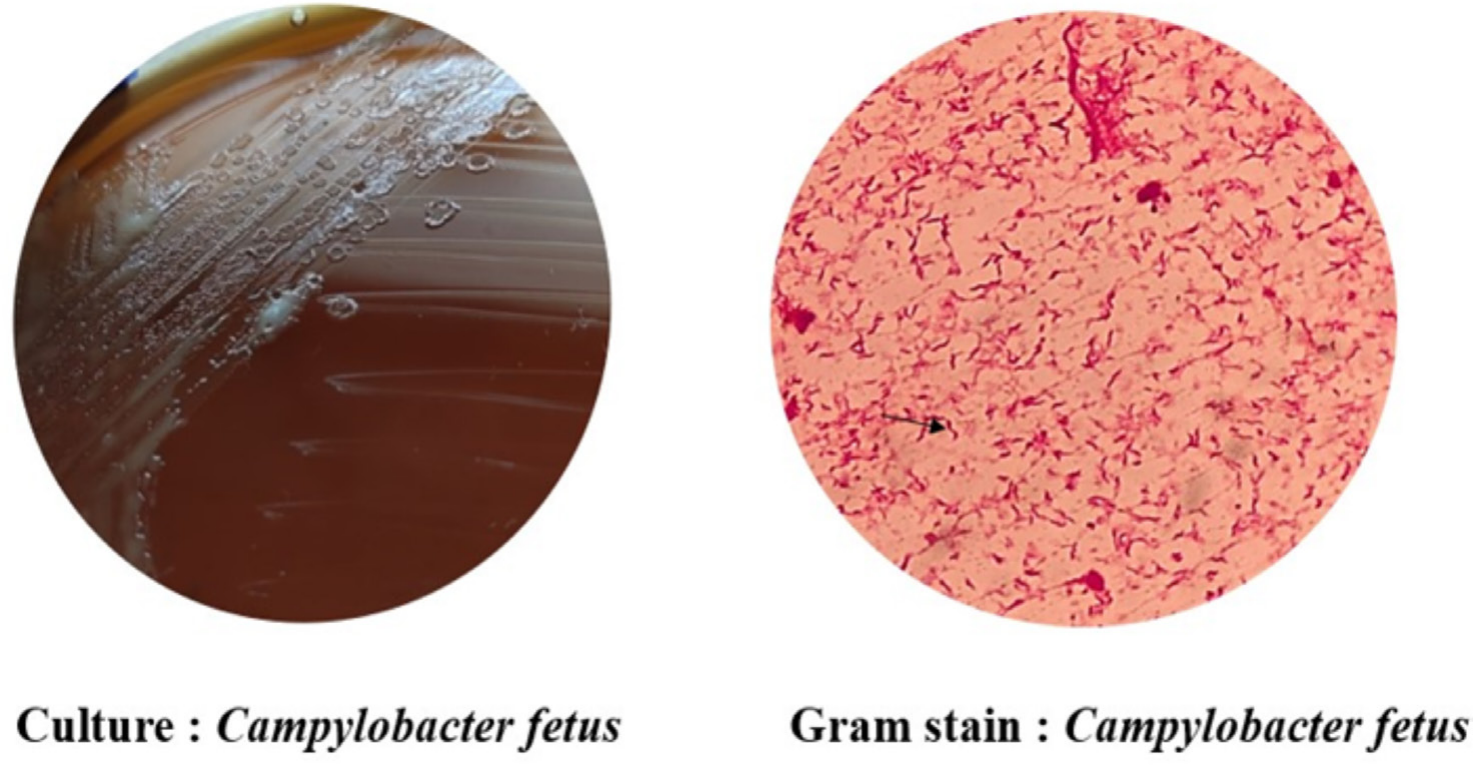
Microaerophilic culture and Gram stain of *Campylobacter fetus* in Campy BAP medium.

The patient was followed up to 6 weeks. In view of the above, the patient was treated for suspected pyogenic spondylodiscitis by starting intravenous cloxacillin 2 g 6 hourly for 1 week. Consequently, after 48 h, scanty growth of fine colonies was seen in a blood agar plate (7 % defibrinated sheep blood used) incubated at 37 °C. A Gram stain showed Gram-negative thin bacilli. A further organism sub-cultured on Campy BAP selective medium under microaerophilic conditions at 42 °C for 24 h showed fine translucent colonies (Fig. 4). A Gram stain performed from the selective media showed Gram-negative thin bacilli with seagull appearance. The colonies were further subjected to matrix-assisted laser desorption/ionization-time of flight mass spectrometry (MALDI-TOF MS) (,VITEK MS version 3.2, bioMérieux, Inc., Durham, NC, USA) and identified as *C. fetus*. Antimicrobial susceptibility testing could not be performed in this study as a viable isolate could not be cultured. After 1 week of treatment, the lower back pain reduced and the patient was able to sit and stand with support. Intravenous cloxacillin was given 2 g 6 hourly for another week. The patient was discharged on day 14 and advised to continue oral antibiotic cloxacillin 1 g 6 hourly for 4 weeks.

The isolate was subsequently subjected to whole-genome sequencing (WGS) to study the rare pathogen characteristics from the unusual site. Genomic DNA was purified from Campy BAP medium using the QIAamp DNA Mini Kit (Qiagen, Hilden, Germany). WGS was performed with 400 bp read chemistry using an IonTorrent Personal Genome Machine (PGM) (Life Technologies, Carlsbad, CA, USA) as per the manufacturer’s instructions. The reads were assembled and annotated using the NCBI Prokaryotic Genome Annotation Pipeline (PGAP). The genomic features of *C. fetus* sequenced in this study are given in Table 1. The species was confirmed as *C. fetus* subsp *fetus* using KmerFinder 3.2 (https://cge.cbs.dtu.dk/services/KmerFinder/) available at the Center for Genomic Epidemiology. Analysis of antimicrobial resistance mechanisms using ResFinder 4.1 (https://cge.cbs.dtu.dk/services/ResFinder/) showed that the isolate does not harbour any acquired genes or point mutation that confers resistance. Notably, mobile genetic elements were not found when screened using MobileElementFinder (https://cge.cbs.dtu.dk/services/MobileElementFinder/). The isolate was also negative for the cytolethal distending toxin (*cdt*A/B/C) gene when analysed. Further, multilocus sequence typing (MLST) analysis using MLSTFinder 2.0 (https://cge.cbs.dtu.dk//services/ MLST/) revealed that the isolate belongs to ST25.

**Table 1.** Genomic data obtained from *Campylobacter fetus* subsp. *fetus* sequenced in this study

Genomic features	*Campylobacter fetus* subsp. *fetus*
Source	Spine sample
BioProject no.	PRJNA663981
Length	1 824 350 bp
Coverage	136x
Contigs	78
Total genes	1909
Total CDS	1861
Total pseudo genes	94
tRNAs	42
rRNAs	3
GC content	33.2
No. of subsystems	195
Virulence factor (VFDB)	87
Resistance (CARD)	4
Sequence type	ST25

## Discussion

In humans, *C. fetus* infection is rare but sometimes fatal. Patients with nonspecific febrile illness who have an occupational risk or are immunocompromised should be suspected of *C. fetus* infection. *C. jejuni* should be considered as a potential cause of systemic illness in differential diagnosis in immunosuppressed patients [7]. Infective spondylodiscitis is a rare musculoskeletal infection associated with high rates of morbidity due to late diagnosis. Diagnosis is usually delayed as the disease is uncommon, with insidious onset of symptoms, and there is difficulty in distinguishing infective spondylodiscitis from degenerative spine disease in elderly adults [8]. This results in longer hospital stays and poorer outcomes. Therefore, the Infectious Diseases Society of America (IDSA) suggested the use of MRI to confirm diagnosis and this remains the gold standard for the radiological demonstration of this condition [8].

Very few studies have documented a case of pyogenic spondylodiscitis caused by *C. fetus* [3]. The pathogenesis of *Campylobacter* infection is complex and still poorly understood. It is believed that all bacterial infections are primarily located in the metaphyseal region of the vertebral body, and that the micro-organism crosses the cartilaginous vertebral plate, runs through the surface of the disc via the anastomotic branches, infects the adjacent vertebral metaphysis, and finally reaches the disc space between the two vertebral bodies involved [9].

In this study, the source of infection and the likely mode of transmission could not be tracked due to patient knowledge gap. No associated immunocompromised condition or systemic illness, such as gastrointestinal tract diseases or bacteraemia, was reported. Generally, in immunocompromised patients, intestinal carriage is a risk factor for bacteraemia, while the disease can be more severe and may persist for long period of time [5, 10]. Indeed, gastroenteritis and bacteraemia can occur in the same patient, at different times [5]. The common underlying conditions in the affected patients included HIV infection, malignancy, liver disease, solid organ transplantation, chronic obstructive pulmonary disease, heart disease and hypogammaglobulinaemia [5]. Studies have also shown that patients having recurrent *Campylobacter* infections indicates their immunodeficient status and this highlights the importance of suspecting hypogammaglobulinaemia [5, 11, 12].

Though erythromycin and fluoroquinolones are the recommended treatment for systemic campylobacteriosis [13], optimal treatment has not yet been defined for *C. fetus* infective spondylodiscitis. However, it is always important to select antibiotics that have good tissue penetration for such infections. Primarily, clindamycin plus ciprofloxacin or cefotaxime plus flucloxacillin are highly recommended to cover a preferably wide spectrum of potential pathogens [14]. In this study, the patient was managed with cloxacillin, given that *S. aureus* is the most common pathogen in non-tubercular cases in India. Although cloxacillin resistance has been reported in *Campylobacter* spp., the study isolate did not harbour any antimicrobial resistance genes. This might be the reason for the better patient outcome. Additionally, percutaneous abscess drainage as an adjunct to traditional treatment would have drastically reduced the focus of infection without the requirement for additional antibiotics. In the case of spondylodiscitis with psoas abscesses, a previous report showed success rates of up to 87.5 % for ultrasound-guided percutaneous drainage placement or CT-guided drainage placement [14].

This implies that accurate laboratory identification of the causative pathogen is required for effectively targeted antimicrobial therapy. Despite this disease being uncommon, it should be remembered that a delay in diagnosis and initiation of treatment may result in instraspinal extension of the infection, with the resultant potential for devastating neurological complications. This report indicates the significance of the causative infectious agent, recalling that *Campylobacter* spp. are difficult to isolate in routine traditional culture. The use of MALDI-TOF in the microbiology laboratory allows rapid and accurate identification of this pathogen. In addition, WGS provides information on the predicted antimicrobial susceptibility of the isolate. This report also highlights the utility of WGS in the identification and characterization of non-viable pathogens, which in turn help the physician switch to more specific treatment regimens for effective management of spondylodiscitis.

### Data availability

The complete genome sequence of *C. fetus* subsp. *fetus* has been deposited in GenBank under the accession number JACXXC000000000.
